# Obesity and low lean mass are associated with dysregulated IGFBP-3, inflammatory biomarkers, and physical impairment in older adult women with frailty

**DOI:** 10.3389/fragi.2026.1765052

**Published:** 2026-02-18

**Authors:** Alan L. Fernandes, Rosa M. R. Pereira, Valeria F. Caparbo, Camila S. Figueredo, Caroline F. Gomes, Eduardo F. B. Neto, Diogo S. Domiciano

**Affiliations:** 1 Rheumatology Division, Hospital das Clinicas HCFMUSP, Faculdade de Medicina da Universidade de Sao Paulo, Sao Paulo, Brazil; 2 Graduate Program in Nursing and Health, School of Nursing, Federal University of Bahia, Salvador, Bahia, Brazil; 3 Acreditando Centro Integrado, Sao Paulo, Brazil

**Keywords:** fat mass, frailty, inflammation, muscle mass, obesity, performance, physical function, sarcopenia

## Abstract

**Introduction:**

Prior studies indicate sex-specific obesity-frailty interactions, with postmenopausal estrogen decline increasing sarcopenic obesity risk and inflammation in women. This study evaluated circulating cytokines (IL-6, TNF-α), adipokines (adiponectin, resistin), myokines (GDF-15, BDNF, myostatin), health-related biomarkers (IGF-1, IGFBP-3), and physical performance (five-times chair stand, grip strength) in pre-frail and frail older adult women classified as having low appendicular lean mass (LALM), obesity, or obesity plus LALM.

**Methods:**

In this cross-sectional study, community-dwelling women aged ≥65 years from São Paulo, Brazil were screened (July 2022–September 2023); among 280 eligible, 88 met Fried frailty criteria. Body composition was assessed by DXA and participants were categorized as LALM (<20th percentile of residuals, −1.45), obesity (body mass index, BMI ≥30 kg/m^2^), or both. Generalized Estimating Equations (GEE) with Bonferroni *post hoc* adjustments, χ^2^, or Fisher’s exact tests were adopted. Unadjusted (P^1^) and age-adjusted (P^2^) P-values were reported.

**Results:**

Among 88 frail women (72.7% pre-frail and 27.3% frail), obesity plus LALM showed lower IGFBP-3 and higher GDF-15 vs. LALM (P^2^ = 0.041 and P^2^ = 0.032); obesity had higher resistin vs. LALM (P^2^ = 0.012), replicated in sensitivity analysis frail-only (P^2^ = 0.002), elevated insulin (P^2^ = 0.002) and a trend slower chair stand (P^2^ = 0.055). GDF-15 was related with chair stand time (Pearson r = 0.285, P = 0.006; and multiple regression β = 0.309, P = 0.013).

**Conclusion:**

Among pre-frail and frail older adult women, obesity—with or without low lean mass—was associated with adverse metabolic/inflammatory profiles (higher resistin, GDF-15, insulin; lower IGFBP-3) in full and frail-only analyses, alongside a trend toward slower chair-stand performance. These cross-sectional findings highlight obesity-frailty interactions, warranting prospective validation.

## 1 Introduction

Aging-related physiological deterioration is accompanied by chronic increase in basal systemic inflammation—inflammaging—with marked endocrine alterations that may imply an increased risk of developing metabolic disorders, termed metainflammation. Both inflammaging and metainflammation contribute towards a profound impairment of health and functional capacity (Dugan et al., 2023). Among the biological systems disrupted with aging, the endocrine axis appears particularly central to the development of frailty (Clegg and Hassan-Smith, 2018), prompting extensive investigation of biomarkers along the aging–frailty continuum (Picca et al., 2022; Moqri et al., 2023; Salvioli et al., 2023). Inflammation plays a pivotal role in this interplay (Vatic et al., 2020; Zhang et al., 2023), and elevated circulating levels of interleukin-6 (IL-6), tumor necrosis factor-alpha (TNF-α), and reduced insulin-like growth factor-1 (IGF-1) have consistently been associated with frailty (Picca et al., 2022; Xu et al., 2022).

More recently, Kim et al. (2025) identified resistin as a promising biomarker of frailty, reporting 52.2% higher circulating concentrations in frail versus robust individuals; each one–standard deviation increases in resistin conferred a 67% higher likelihood of frailty, and individuals in the highest quartile had over 12-fold greater odds of being frail compared with those in the lowest quartile. Together, these findings underscore the centrality of immunometabolic dysregulation in frailty pathophysiology.

Frailty is a multifactorial clinical syndrome characterized by unintentional weight loss, weakness, slowness, and exhaustion (Fried et al., 2001), and it often overlaps with sarcopenia. Chew et al. (2019) demonstrated that among frail older adults, those with concomitant low appendicular lean mass (ALM) exhibited elevated standardized myostatin levels and lower IGF-1 compared with frail individuals with preserved ALM—associations not observed in robust participants. These observations suggest shared biological pathways between sarcopenia and frailty, consistent with the heightened risk of recurrent falls, fractures, and functional decline reported in individuals presenting both deficits (Lee D. et al., 2022).

Prior studies indicate sex-specific obesity-frailty interactions (Gordon et al., 2017), with postmenopausal estrogen decline increasing sarcopenic obesity risk and inflammation in women (Geraci et al., 2021). Chronic low-grade inflammation also contributes to obesity (Hotamisligil, 2017), characterized by elevated pro-inflammatory cytokines (Frasca et al., 2017), which may link frailty, sarcopenia, and obesity (Buch et al., 2016; Kalinkovich and Livshits, 2017; Livshits and Kalinkovich, 2019). Despite recognition of these interconnected processes, circulating immunometabolic markers—including cytokines, adipokines, and myokines—remain poorly draw in older adults with frailty who exhibit distinct patterns of adverse body composition.

Given this gap in the literature, the present study aimed to evaluate inflammatory and metabolic biomarkers, health-related variables, and physical performance across three body-composition phenotypes—low appendicular lean mass (LALM), obesity, and their coexistence—in pre-frail and frail community-dwelling older women. We hypothesized that individuals presenting both obesity and LALM would exhibit the most unfavorable biomarker profiles and the greatest impairment in physical function, reflecting the cumulative burden of dual deficits in muscle and adiposity.

## 2 Methods

### 2.1 Study design and participants

This cross-sectional study included community-dwelling older adult women from São Paulo, Brazil, between July 2022 and September 2023. The protocol was approved by the Ethics Committee of the Hospital das Clínicas, School of Medicine, University of São Paulo (HC-FMUSP, CAAE: 31958520.4.0000.0068) and conducted in accordance with the Declaration of Helsinki and Good Clinical Practice guidelines. All participants provided written informed consent.

Eligible individuals were women aged ≥65 years. Participants were recruited via geriatrics outpatient clinics (HC-FMUSP), prior study registries, and direct community referrals. Exclusion criteria included a diagnosis of dementia, physical disability, use of insulin, glucocorticoids, or medications affecting muscle function or bone mass; chronic obstructive pulmonary disease (COPD) or chronic kidney disease; uncontrolled chronic non-communicable diseases (including diabetes, hypertension, dyslipidemia, thyroid disorders, or liver disease); restrictive diets; or recent use (within the prior 12 months) of bisphosphonates, denosumab, teriparatide, or romosozumab. All those who did not meet the exclusion criteria were assessed for frailty.

### 2.2 Frailty phenotype

Frailty was assessed according to the Fried frailty phenotype (2001), which evaluates unintentional weight loss (≥4.55 kg or >5% body weight in the preceding year), self-reported exhaustion, weakness (handgrip strength), slow walking speed (4.6 m), and low physical activity. Participants were categorized as robust (no criteria), pre-frail (1–2 criteria), or frail (≥3 criteria). Only pre-frail and frail community-dwelling older women were included.

### 2.3 Clinical assessment

A standardized face-to-face questionnaire collected demographic and clinical data, including age, comorbidities, medication use, smoking status, alcohol intake, supplementation, and physical activity. Anthropometric measurements (height, weight, and body mass index—BMI) were obtained according to standard procedures to confirm self-reported information. Obesity was defined as BMI ≥30 kg/m^2^ (WHO, 1997).

### 2.4 Laboratory data

Fasting blood samples (8-h fast) were collected between 7:30 and 9:30 a.m. Measurements included glycated hemoglobin, insulin, very low-density lipoprotein (VLDL), LDL-cholesterol, HDL-cholesterol, total cholesterol, triglycerides, ferritin, and erythrocyte sedimentation rate, analyzed using automated standardized methods in an accredited hospital laboratory.

### 2.5 Body composition

Body composition was evaluated by whole-body dual-energy X-ray absorptiometry (DXA) using a Lunar iDXA scanner (GE Healthcare). Total fat mass, appendicular lean mass (ALM), and total lean mass were assessed. ALM was defined as the sum of lean soft tissue in both upper and lower limbs (Siervo et al., 2015) and used to classify low ALM (LALM) according to fat-adjusted criteria previously applied in the São Paulo Aging and Health (SPAH) Study (Lopes et al., 2011).

### 2.6 Definition of low appendicular lean mass (LALM)

Low appendicular lean mass (LALM) was defined using São Paulo Ageing and Health Study (SPAH) criteria from prior Brazilian studies (Domiciano et al., 2013; Figueiredo et al., 2014). Expected ALM was calculated via the linear regression model proposed by Newman et al. (2003), which incorporates height (m^2^) and total fat mass (kg). The residual (measured ALM minus expected ALM) reflects deviation from predicted muscle mass: positive values denote higher-than-expected ALM, whereas negative values indicate lower ALM.

For women, the model equation is: *ALM (kg) = −14.51 + 17.27 × height (m) + 0.20 × fat mass (kg).* The 20th percentile of the residual distribution (−1.45) was used as the cutoff for LALM in Brazilian older women (Domiciano et al., 2013).

### 2.7 Physical performance

Lower-extremity function was assessed using the five-times chair stand test, in which participants are timed while rising from and sitting back down on a chair five consecutive times without using their arms (Pavasini et al., 2016).

Handgrip strength was measured with a Jamar® dynamometer (Lafayette Instrument Company, United States). Participants performed three trials on each hand, alternating sides with one-minute intervals. The highest value obtained from the dominant hand was used for analysis (Roberts et al., 2011).

Chair stand and grip strength were selected as direct, frailty-sensitive measures aligned with Fried criteria (Suzuki et al., 2023; Kawamura et al., 2025). Bohannon (2019) reiterates grip strength as a biomarker for identifying at-risk older adults, while Johansson et al. (2023) demonstrate their independent mortality associations, supporting combined rather than interchangeable use.

### 2.8 Cytokines, adipokines, myokines, and health-related markers

Cytokines (IL-6, TNF-α), adipokines (adiponectin, resistin), and myokines [growth differentiation factor-15 (GDF-15), brain-derived neurotrophic factor (BDNF), and myostatin] were quantified using the Luminex® xMAP multiplex platform with commercial Milliplex MAP kits (HAGE1MAG-20K, HADCYMAG-61K, HMYOMAG-56K; Millipore Corp., Billerica, MA, United States). All assays were run simultaneously by a blinded technician following manufacturer instructions.

Serum IGF-1 and IGFBP-3 were measured via chemiluminescence in an accredited clinical laboratory. Intra-assay coefficients of variation were: GDF-15 (1.51%), IL-6 (1.41%), TNF-α (1.17%), adiponectin (0.98%), resistin (2.84%), BDNF (0.69%), and myostatin (0.63%).

### 2.9 Statistical analysis

Sample size estimation was based on skeletal muscle index (SMI, kg/m^2^) differences between older adults with obesity versus sarcopenia (Fukuda et al., 2018), yielding a required sample of 44 participants to achieve 95% power at α = 0.05 using G*Power (version 3.1.2).

Normality assumptions were verified prior to analyses. Generalized Estimating Equations (GEE) with independent working correlation structure was adopted, providing robust standard errors appropriate for unbalanced cross-sectional data. Both unadjusted (P^1^) and age-adjusted (P^2^) P-values are reported for the group main effect. Bonferroni *post hoc* testes, estimated marginal means (EMMEANS), provide pairwise unadjusted (Bonferroni *post hoc* P^1^) and age-adjusted (Bonferroni *post hoc* P^2^) P-values for multiple group comparisons, using a two-tailed significance threshold of 0.05.

Categorical variables were evaluated with χ^2^ or Fisher’s exact tests. Pearson correlation coefficients were computed separately for each dependent variable—chair stand time (seconds) and handgrip strength (kg)—against cytokines, adipokines, myokines, and health-related markers as independent variables. Multiple linear regressions were then performed separately for each dependent variable (chair stand time, handgrip strength and body composition parameters), adjusting for age. Full model specifications, including β coefficients, unstandardized B with 95% CIs, and P-values were also reported.

Data are presented as mean ± SD, median (interquartile range), or n (%). Statistical analyses were conducted using IBM SPSS Statistics version 26.0.

## 3 Results

Of the 280 eligible participants, 88 met frailty criteria [64 pre-frail (72.7%), and 24 frail (27.3%)], with mean ± SD age 72.8 ± 4.8 years and BMI 29.9 ± 5.8 kg/m^2^. Participants were classified into three phenotypes: LALM (n = 47), obesity (n = 21), and obesity plus LALM (n = 20) (Table 1). Total lean mass was significantly lower in the LALM group compared with both the obesity (35.2 ± 3.1 vs. 44.5 ± 5.3 kg, Bonferroni *post hoc* P < 0.001 for both P^1^ and P^2^) and obesity plus LALM groups (vs. 38.6 ± 4.3 kg, Bonferroni *post hoc* P^1^ = 0.003 and P^2^ = 0.004). The obesity group also exhibited higher total lean mass than the obesity plus LALM group (mean difference ±SE: 5.9 ± 1.5 kg, Bonferroni *post hoc* P < 0.001 for both). Conversely, total fat mass and BMI were markedly lower in the LALM group (27.4 ± 5.5 kg and 25.7 ± 2.9 kg/m^2^) compared with both the obesity (39.6 ± 9.9 kg and 35.4 ± 5.2 kg/m^2^, Bonferroni *post hoc* P < 0.001 for all) and the obesity plus LALM groups (39.5 ± 7.5 kg and 34.0 ± 3.8 kg/m^2^, Bonferroni *post hoc* P < 0.001 for all). Fat mass and BMI did not differ significantly between the two obesity phenotypes in pairwise comparisons of the Bonferroni *post hoc* test. All findings remained statistically consistent after age adjustment (Table 1).

**TABLE 1:** Demographic and anthropometric characteristics, body composition, laboratory parameters, comorbidities and functional performances per group.

Characteristic	LALM group (n = 47)	Obesity group (n = 21)	Obesity plus LALM group (n = 20)	P^1^	P^2^
Age, years	73.0 ± 5.3	72.7 ± 4.5	72.4 ± 4.3	0.897	-
Body weight, kg	64.5 ± 7.9^a^	86.9 ± 14.4^b^	81.2 ± 11.8^b^	**<0.001**	**<0.001**
Body mass index, kg/m^2^	25.7 ± 2.9^a^	35.4 ± 5.2^b^	34.0 ± 3.8^b^	**<0.001**	**<0.001**
Total lean mass, kg	35.2 ± 3.1^a^	44.5 ± 5.3^b^	38.6 ± 4.3^c^	**<0.001**	**<0.001**
Total fat mass, kg	27.4 ± 5.5^a^	39.6 ± 9.9^b^	39.5 ± 7.5^b^	**<0.001**	**<0.001**
Pre-frail, n (%)	38 (43.2)	13 (14.8)	13 (14.8)	0.213	-
Frail, n (%)	9 (10.2)	8 (9.1)	7 (7.9)		
Laboratory parameters					
Glycated hemoglobin, %	5.6 ± 0.5	5.8 ± 0.6	6.1 ± 0.9	0.064	**0.046**
Insulin, µU/mL	8.4 ± 3.9^a^	13.6 ± 6.8 ^b^	12.0 ± 5.7 ^b^	**<0.001**	**<0.001**
VLDL-cholesterol, mg/dL	22.0 ± 5.9	24.3 ± 10.9	23.2 ± 7.1	0.582	0.594
LDL-cholesterol, mg/dL	128.2 ± 37.9	122.9 ± 37.4	130.0 ± 56.1	0.832	0.825
HDL-cholesterol, mg/dL	60.2 ± 11.1	58.2 ± 13.4	57.9 ± 17.9	0.758	0.771
Cholesterol, mg/dL	206.0 ± 48.0	205.4 ± 45.0	211.0 ± 64.4	0.943	0.961
Triglycerides, mg/dL	111.5 ± 41.7	131.6 ± 80.6	120.8 ± 47.9	0.455	0.458
Ferritin, ng/mL	156.4 ± 92.3	166.4 ± 113.7	122.6 ± 77.7	0.225	0.238
Erythrocyte sedimentation rate, mm	15.0 ± 11.3	12.8 ± 10.6	14.8 ± 16.0	0.718	0.726
Coexisting diseases, n (%)					
Diabetes	6 (12.8)	6 (28.6)	4 (20.0)	0.281	-
Hypertension	28 (59.6)	12 (57.1)	12 (60.0)	1.000	-
Thyroid dysfunction	20 (42.6)	7 (33.3)	9 (45.0)	0.711	-
Dyslipidemia	17 (36.2)	8 (38.1)	10 (50.0)	0.595	-
Depression	6 (12.8)	3 (14.3)	3 (15.0)	1.000	-
Anxiety	1 (2.1)	2 (9.5)	3 (15.0)	0.093	-
Physical performance					
Five-times chair stand, sec	11.8 ± 2.9^a^	15.4 ± 7.0 ^b^	13.3 ± 7.5^a,b^	**0.051**	**0.046**
Handgrip strength, kg	19.4 ± 3.9	21.8 ± 4.8	18.9 ± 4.7	0.091	0.101

Values are mean ± SD, or n (%). Continuous variables were analyzed by Generalized Estimating Equations (GEE) with independent working correlation structure. Percentages were analyzed by chi-square or Fisher’s exact test. Both unadjusted (P^1^) and age-adjusted (P^2^) P-values are reported for the group main effect. Values in bold mean statistical difference. Different superscripts (a, b, c) represent statistically significant differences between groups from the age-adjusted Bonferroni *post hoc* test (i.e., Bonferroni *post hoc* P^2^). Groups that share at least one superscript letter do not differ significantly. In the five-times chair stand test, the LALM, group tended to be faster only compared to the obesity group (11.8 ± 2.9 vs. 15.4 ± 7.0 s, Bonferroni *post hoc* P^1^ = 0.058 and P^2^ = 0.055). The other pairwise comparisons did not differ (P > 0.05).

Insulin concentrations were significantly lower in the LALM group compared with both the obesity (8.4 ± 3.9 vs. 13.6 ± 6.8 µU/mL, Bonferroni *post hoc* P^1^ = 0.003 and P^2^ = 0.002) and obesity plus LALM groups (vs. 12.0 ± 5.7 µU/mL, Bonferroni *post hoc* P^1^ = 0.028; P^2^ = 0.018). Five-times chair stand performance showed a significant difference for the group main effect in both unadjusted (P^1^ = 0.051) and age-adjusted (P^2^ = 0.046) models (Table 1); although Bonferroni *post hoc* test exhibited a trend for the LALM group to be faster than the obesity group (11.8 ± 2.9 vs. 15.4 ± 7.0 s, Bonferroni *post hoc* P^1^ = 0.058 and P^2^ = 0.055). No significant differences were observed for insulin or chair stand performance among the other phenotypes in pairwise comparisons.

**FIGURE 1:**
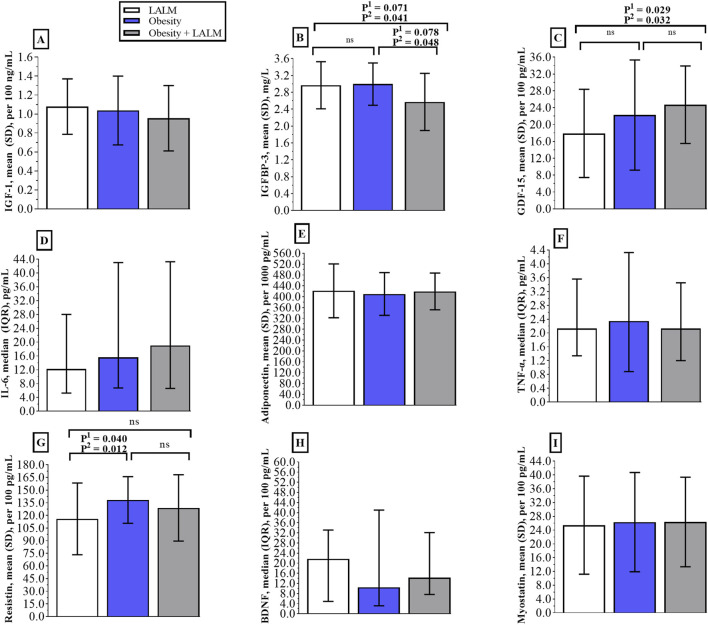
Plasma concentrations of IGF-1 **(A)**, IGFBP-3 **(B)**, GDF-15 **(C)**, IL-6 **(D)**, adiponectin **(E)**, TNF-alfa **(F)**, resistin **(G)**, BDNF **(H)**, and myostatin **(I)** among groups. The white, blue, and gray bars represent the LALM, Obesity, and Obesity plus LALM groups, respectively. Values are mean ± SD or median (IQR). IGF-1, insulin-like growth factor 1; IGFBP-3, insulin-like growth factor binding protein 3; GDF-15, growth differentiating factor 15; IL-6, interleukin 6; TNF-α, tumor necrosis factor alpha; BDNF, brain-derived neurotrophic factor. Continuous variables were analyzed by Generalized Estimating Equations (GEE) with independent working correlation structure. P1 value represents the unadjusted Bonferroni post-hoc test in the pairwise comparison. P2 value represents the age-adjusted Bonferroni post-hoc test in the pairwise comparison.

IGFBP-3 concentrations showed a group main effect of marginal significance in the unadjusted model (P^1^ = 0.054) and statistical significance in the age-adjusted model (P^2^ = 0.034) models. The obesity plus LALM group showed borderline significantly lower IGFBP-3 levels than both the obesity (2.6 ± 0.7 vs. 3.0 ± 0.5 mg/L, Bonferroni *post hoc* P^1^ = 0.078 and P^2^ = 0.048) and LALM groups (vs. 3.0 ± 0.6 mg/L, Bonferroni *post hoc* P^1^ = 0.071 and P^2^ = 0.041) (Figure 1). IGFBP-3 levels were similar between the obesity and LALM groups. GDF-15 concentrations showed a significant group main effect in both unadjusted (P^1^ = 0.030) and age-adjusted (P^2^ = 0.032) models. GDF-15 concentrations were significantly higher in the obesity plus LALM group than in the LALM group (24.7 ± 9.2 vs. 17.9 ± 10.4 per 100 pg/mL, Bonferroni *post hoc* P^1^ = 0.029 and P^2^ = 0.032). Similarly, resistin concentrations showed a significant group main effect in both unadjusted (P^1^ = 0.047) and age-adjusted (P^2^ = 0.016) models. Resistin was significantly higher in the obesity group compared with the LALM group (138.3 ± 27.7 vs. 115.9 ± 42.5 per 100 pg/mL, Bonferroni *post hoc* P^1^ = 0.040 and P^2^ = 0.012). All other pairwise comparisons for GDF-15 and resistin were nonsignificant, and no differences were detected across groups for IGF-1, IL-6, adiponectin, TNF-α, BDNF, or myostatin (Figure 1).

In the sensitivity analysis restricted to frail participants only (≥3 Fried criteria), resistin concentrations showed a significant group main effect in both unadjusted (P^1^ = 0.018) and age-adjusted (P^2^ = 0.003) models. Post-hoc Bonferroni comparisons revealed significantly higher resistin levels in the obesity group versus LALM group (143.0 ± 18.9 vs. 113.6 ± 28.0 pg/mL; P^1^ = 0.023, P^2^ = 0.002). All other pairwise resistin comparisons and group effects for five-times chair stand, handgrip strength, IGF-1, IGFBP-3, GDF-15, IL-6, adiponectin, TNF-α, BDNF, and myostatin were nonsignificant.

In the full cohort, GDF-15 demonstrated a weak but significant positive correlation with time to complete the five-times chair stand test (Pearson r = 0.285, P = 0.006) and remained associated in the age-adjusted model (β = 0.309, P = 0.013) (Table 2). No other cytokines, adipokines, myokines, or metabolic markers were significantly associated with chair stand or grip strength performance. Pearson correlation coefficients and multiple linear regressions were performed separately for each physical performance outcome (chair stand time, handgrip strength) by group, and body composition parameters (lean mass, ALM, fat mass and % fat) as dependent variable in the total sample in the Supplementary Material (Supplementary Tables S1–S4).

**TABLE 2:** Pearson correlation coefficients and multiple linear regression analysis of physical performance, cytokines, adipokines, myokines, and health-related markers assessed in total sample.

Pearson correlation	Five-times chair stand, sec		Handgrip strength, kg	
	*r*	P	*r*	P
GDF-15, pg/mL	0.285	**0.006**	0.024	0.417
IL-6, pg/mL	0.016	0.445	−0.090	0.216
Adiponectin, pg/mL	0.048	0.341	0.115	0.159
TNF-alfa, pg/mL	0.026	0.413	0.041	0.360
Resistin, pg/mL	0.014	0.454	0.080	0.243
BDNF, pg/mL	−0.007	0.478	0.064	0.289
Myostatin, pg/mL	0.020	0.432	−0.081	0.244
IGF-1, ng/mL	−0.121	0.135	0.088	0.210
IGFBP-3 mg/L	0.049	0.329	0.156	0.076

Multiple linear regression	ß	B	(95% CI)	P	ß	B	(95% CI)	P
GDF-15, pg/mL	0.309	0.002	(0.000 to 0.003)	**0.013**	−0.009	−3.542^e−5^	(−0.001 to 0.001)	0.942
IL-6, pg/mL	0.045	0.004	(−0.024 to 0.033)	0.769	−0.175	−0.013	(−0.036 to 0.010)	0.262
Adiponectin, pg/mL	0.027	1.759^e−6^	(0.000 to 0.000)	0.826	0.149	7.611^e−6^	(0.000 to 0.000)	0.245
TNF-alfa, pg/mL	0.008	0.006	(−0.233 to 0.245)	0.959	0.173	0.110	(−0.085 to 0.304)	0.263
Resistin, pg/mL	−0.077	0.000	(0.000 to 0.887)	0.534	0.112	0.000	(0.000 to 0.000)	0.376
BDNF, pg/mL	0.027	5.820^e−5^	(0.001 to 0.798)	0.839	0.106	0.000	(0.000 to 0.001)	0.429
Myostatin, pg/mL	−0.049	0.000	(0.001 to 0.698)	0.727	−0.123	0.000	(−0.001 to 0.001)	0.390
IGF-1, ng/mL	−0.286	−0.049	(0.007 to 0.513)	0.084	−0.028	−0.004	(−0.049 to 0.041)	0.865
IGFBP-3 mg/L	0.214	1.997	(4.850 to 0.577)	0.167	0.139	1.034	(−1.285 to 3.352)	0.376

Values in bold mean statistical difference.

## 4 Discussion

The present study demonstrates that obesity—either alone or in combination with low appendicular lean mass—was associated with an adverse biomarker profile characterized by reduced IGFBP-3, elevated resistin, higher GDF-15, greater insulin concentrations, and impaired lower-extremity performance in pre-frail and frail community-dwelling older women. Resistin was the sole biomarker with significant group effects in both full and frail-only samples, remaining significant after Bonferroni correction (obesity vs. LALM). All other biomarkers showed nonsignificant results (P > 0.05). To our knowledge, this is the first study to directly compare distinct adverse body composition phenotypes (LALM, obesity, and obesity plus LALM) within a sample exclusively with frailty, providing novel evidence regarding the intrinsic inflammatory and metabolic heterogeneity characteristic of this syndrome.

### 4.1 IGFBP-3: linking adiposity, muscle loss and inflammation in frailty

The markedly lower IGFBP-3 levels observed in the obesity plus LALM group align with findings by Gomez et al. (2004), who reported reduced IGFBP-3 in women with higher BMI and fat mass. IGFBP-3 plays a dual metabolic and anti-inflammatory role—preserving muscle homeostasis, modulating IGF-1 signalling, and exerting protective effects against obesity-related inflammation (Palau et al., 2012; Mohanraj et al., 2013). Individuals presenting both obesity and low muscle mass exhibit two overlapping metabolic stressors: elevated adiposity (Kim, 2013) and reduced muscle anabolic capacity (Ferrari et al., 2021). This combined phenotype may therefore deplete IGFBP-3 as part of a broader catabolic–inflammatory response, consistent with its association with multiple age-related diseases such as cognitive impairment, neurodegeneration, and osteoporosis (Yang et al., 2005; Wennberg et al., 2018).

Furthermore, experimental evidence suggests that IGFBP-3 may mitigate obesity-induced insulin resistance (Mohanraj et al., 2013), reinforcing the plausibility of the more insulin-resistant profile observed in the obesity and obesity plus LALM groups in our study.

### 4.2 Resistin: a marker of metabolic stress and frailty burden

Resistin levels were significantly higher in individuals with obesity, including in the sensitivity analysis restricted to frail participants, consistent with prior studies linking resistin to chronic inflammation, metabolic dysfunction, frailty, sarcopenia, and all-cause mortality in older adults (Khan et al., 2014; Parkkila et al., 2021; Kwak et al., 2024; Kim et al., 2025). Kim et al. (2025) recently identified resistin as a promising biomarker of frailty, reporting markedly elevated levels in frail individuals; notably, each 1-SD increase in resistin was associated with a 67% higher risk of frailty. Our findings extend this concept by showing that, even within a frailty-enriched sample, the metabolic signature of obesity is associated with higher resistin concentrations. This supports the notion that inflammatory adipokines may help discriminate frailty phenotypes with distinct metabolic risks, even when clinical frailty is already established.

### 4.3 GDF-15: a stress cytokine bridging adiposity, muscle catabolism and physical decline

GDF-15 has emerged as a robust biomarker of aging, systemic stress, mitochondrial dysfunction, and muscle catabolism. Elevated GDF-15 has been linked to frailty (Cardoso et al., 2018; Arauna et al., 2020; Suganuma et al., 2024), physical impairment (Tavenier et al., 2021), and pain-related disability (Tarabeih et al., 2019), and our findings confirm higher circulating GDF-15 in participants with obesity plus LALM. The literature supports a strong association between GDF-15 and unfavourable body composition phenotypes, including sarcopenia, obesity, osteosarcopenic obesity, and their combinations (Lee S. H. et al., 2022).

Mechanistically, GDF-15 promotes anorexigenic signalling, muscle wasting pathways, and reduced energetic capacity—potentially accelerating functional decline, especially in individuals with coexisting muscle and fat abnormalities. The observed positive correlation between GDF-15 and slower five-times chair stand performance strengthens the hypothesis that GDF-15 may serve as a mediator linking metabolic stress to impaired physical function in frailty. Similar associations were reported by Newman et al. (2016) in non-frail older adults, suggesting that this mechanism persists—or may even be amplified—in frail populations.

### 4.4 Functional performance: a converging endpoint of inflammation and adverse body composition

Both the obesity and obesity plus LALM groups required more time to complete the five-times chair stand test, indicating reduced lower-extremity functional capacity, although the difference reached statistical significance only in the comparison between the obesity and LALM groups. Poor physical performance has long been associated with elevated circulating cytokines in older adults, including slower chair stand time, lower SPPB scores (Kwak et al., 2024), and reduced gait speed (Wennberg et al., 2018). Consistent with these findings, we observed in this pre-frail and frail cohort significant positive associations between GDF-15 and chair stand duration (both Pearson r = 0.285, P = 0.006 and β = 0.309, P = 0.013), supporting the notion that GDF-15 may contribute to inflammation-related declines in muscle function, as previously described by Newman et al. (2016) in non-frail older adults.

From a clinical perspective, both obesity (15.4 s) and obesity plus LALM (13.3 s) groups exceeded the reference threshold of 12.6 s proposed by Bohannon (2006), reinforcing the functional impairment associated with these phenotypes. These values surpassed established cutoffs linked to increased fall risk (≥12 s) (Tiedemann et al., 2008) and, in the obesity group, approached the threshold associated with recurrent falls (>15 s) (Buatois et al., 2010). Taken together, these findings suggest that adiposity-related inflammatory stress is associated with greater lower-limb functional decline even within a population that already has frailty.

### 4.5 Interpretation and implications

LALM adoption followed Domiciano et al. (2013), demonstrating that traditional ALM/height^2^ cutoffs did not accurately discriminate sarcopenia in overweight/obese community-dwelling Brazilian older adult women. International consensus (EWGSOP2, AWGS, FNIH) endorses population-specific thresholds due to ethnic/anthropometric variability. Therefore, it was plausible to adopt regression models adjusted ALM for fat mass/height for enhanced LALM accuracy over rigid cutoffs.

Frailty is not a homogeneous inflammatory state, as inflammaging exhibits individualized expression modulated by body composition phenotypes. In our study, obesity plus LALM was associated with suppressed IGFBP-3, elevated GDF-15, and a trend towards slower chair-stand performance—potentially representing a high-risk phenotype among heterogeneous frailty presentations, warranting personalized phenotyping approaches.

This underscores the need to integrate body composition profiling—particularly DXA-derived parameters—with biomarker assessment when evaluating frailty risk, prognosis, and potential therapeutic targets. In clinical practice, these findings highlight that pre-frail and frail older women with concomitant obesity and low muscle mass may benefit from interventions targeting both muscle anabolism and adiposity-related inflammation.

### 4.6 Strengths and limitations

Robust methodological controls minimized Type I errors (false positives) across multiple biomarker comparisons, including sample size exceeding requirements for 95% power (n = 44); GEE analyses (vs. GLM) accounting for data correlation; low intra-assay CVs (<3%, range 0.63%–2.84%) via Luminex® assays; and frail-only sensitivity analyses confirmed key resistin findings. All P-values are transparently reported with null findings labelled exploratory, enhancing interpretative caution. This approach acknowledges statistical uncertainties in exploratory biomarker research on inflammaging across obesity-frailty phenotypes in an understudied cohort.

Limitations include female-only Brazilian sample limits generalizability; cross-sectional design precludes causality; formal sarcopenia subanalysis (ALM < 15 kg or ALM/h^2^ < 5.5 kg/m^2^) identified only 3 cases, precluding group comparisons; lack of visceral/regional fat distribution assessment. Longitudinal studies with comprehensive fat/muscle phenotyping are needed to establish causality.

## 5 Conclusion

Obesity, alone or combined with low appendicular lean mass, appears associated with an altered inflammatory and metabolic profile—including reduced IGFBP-3, higher resistin and GDF-15, increased insulin concentrations, and a trend towards slower chair-stand performance—in pre-frail and frail older adult women. These findings underscore frailty heterogeneity and highlight the importance of considering body composition phenotypes to identify subgroups at heightened risk for accelerated functional decline and adverse health outcomes.

## Data Availability

The original contributions presented in the study are included in the article/Supplementary Material, further inquiries can be directed to the corresponding author.

## In memoriam

In memory of author Rosa M. R. Pereira.
