# Supplementary material for: Obesity and low lean mass are associated with dysregulated IGFBP-3, inflammatory biomarkers, and physical impairment in older adult women with frailty
Source: Front Aging. 2026 Feb 18;7:1765052. doi: 10.3389/fragi.2026.1765052 (PMC12957219; doi:10.3389/fragi.2026.1765052)
Supplement: Supplementary file 1 [file Table1.docx]

Supplementary Material

# Supplementary Table 1. Pearson correlation coefficient and multiple linear regression analyses examining functional performance, cytokines, adipokines, myokines, and health-related markers in participants with LALM.

|  | **5x-Chair stand, sec** | | | | **Handgrip strength, kg** | | | |
| --- | --- | --- | --- | --- | --- | --- | --- | --- |
| *Pearson correlation* |  | ***r*** | **P** |  |  | ***r*** | **P** |  |
| GDF-15, pg/mL |  | 0.288 | **0.040** |  |  | 0.108 | 0.256 |  |
| IL-6, pg/mL |  | -0.263 | 0.056 |  |  | -0.170 | 0.150 |  |
| Adiponectin, pg/mL |  | 0.053 | 0.376 |  |  | 0.097 | 0.279 |  |
| TNF-alfa, pg/mL |  | -0.198 | 0.117 |  |  | -0.012 | 0.471 |  |
| Resistin, pg/mL |  | 0.214 | 0.099 |  |  | -0.396 | **0.006** |  |
| BDNF, pg/mL |  | -0.285 | **0.041** |  |  | 0.148 | 0.184 |  |
| Myostatin, pg/mL |  | -0.210 | 0.109 |  |  | 0.101 | 0.276 |  |
| IGF-1, ng/mL |  | -0.350 | **0.009** |  |  | 0.172 | 0.124 |  |
| IGFBP-3 mg/L |  | -0.113 | 0.227 |  |  | 0.172 | 0.124 |  |
| *Multiple linear regression* | **ß** | **B** | **(95% CI)** | **P** | **ß** | **B** | **(95% CI)** | **P** |
| GDF-15, pg/mL | 0.188 | 0.001 | (0.000 to 0.001) | 0.283 | 0.233 | 0.001 | (-0.001 to 0.001) | 0.204 |
| IL-6, pg/mL | -0.153 | -0.009 | (-0.034 to 0.016) | 0.464 | -0.327 | -0.026 | (-0.036 to 0.010) | 0.139 |
| Adiponectin, pg/mL | 0.180 | 5.328^e-6^ | (0.000 to 0.000) | 0.353 | 0.027 | 1.059^e-6^ | (0.000 to 0.000) | 0.894 |
| TNF-alfa, pg/mL | -0.084 | -0.031 | (-0.187 to 0.145) | 0.682 | 0.210 | 0.105 | (-0.085 to 0.304) | 0.333 |
| Resistin, pg/mL | 0.067 | 4.566^e-5^ | (0.000 to 0.000) | 0.689 | -0.423 | 0.000 | (0.000 to 0.000) | **0.021** |
| BDNF, pg/mL | -0.218 | 0.000 | (-0.001 to 0.000) | 0.289 | 0.041 | 9.170^e-5^ | (0.000 to 0.001) | 0.845 |
| Myostatin, pg/mL | -0.194 | 0.000 | (-0.001 to 0.001) | 0.412 | 0.085 | 0.000 | (-0.001 to 0.001) | 0.728 |
| IGF-1, ng/mL | -0.431 | -0.043 | (-0.086 to 0.001) | 0.057 | 0.157 | 0.021 | (-0.049 to 0.041) | 0.493 |
| IGFBP-3 mg/L | 0.156 | 0.804 | (-1.434 to 3.042) | 0.467 | -0.008 | -0.058 | (-1.285 to 3.352) | 0.970 |

Values in bold mean statistical difference.

**Supplementary Table S2. Pearson correlation coefficient and multiple linear regression analyses examining functional performance, cytokines, adipokines, myokines, and health-related markers in participants with obesity.**

|  | **5x-Chair stand, sec** | | | | **Handgrip strength, kg** | | | |
| --- | --- | --- | --- | --- | --- | --- | --- | --- |
| *Pearson correlation* |  | ***r*** | **P** |  |  | ***r*** | **P** |  |
| GDF-15, pg/mL |  | 0.421 | **0.032** |  |  | -0.133 | 0.288 |  |
| IL-6, pg/mL |  | 0.055 | 0.409 |  |  | -0.202 | 0.197 |  |
| Adiponectin, pg/mL |  | 0.418 | **0.033** |  |  | 0.197 | 0.225 |  |
| TNF-alfa, pg/mL |  | 0.006 | 0.489 |  |  | 0.013 | 0.479 |  |
| Resistin, pg/mL |  | -0.013 | 0.479 |  |  | 0.215 | 0.182 |  |
| BDNF, pg/mL |  | -0.098 | 0.340 |  |  | -0.204 | 0.195 |  |
| Myostatin, pg/mL |  | 0.018 | 0.471 |  |  | -0.291 | 0.106 |  |
| IGF-1, ng/mL |  | 0.021 | 0.465 |  |  | -0.132 | 0.285 |  |
| IGFBP-3 mg/L |  | 0.226 | 0.169 |  |  | -0.063 | 0.395 |  |
| *Multiple linear regression* | **ß** | **B** | **(95% CI)** | **P** | **ß** | **B** | **(95% CI)** | **P** |
| GDF-15, pg/mL | 0.373 | 0.002 | (-0.002 to 0.006) | 0.283 | -0.413 | -0.002 | (-0.004 to 0.001) | 0.231 |
| IL-6, pg/mL | 0.279 | 0.024 | (-0.049 to 0.096) | 0.488 | -0.530 | -0.031 | (-0.080 to 0.019) | 0.196 |
| Adiponectin, pg/mL | 0.265 | 2.326^e-5^ | (0.000 to 0.000) | 0.410 | 0.410 | 2.472^e-5^ | (0.000 to 0.000) | 0.206 |
| TNF-alfa, pg/mL | -0.429 | -0.392 | (-1.304 to 0.519) | 0.360 | 0.317 | 0.199 | (-0.418 to 0.816) | 0.489 |
| Resistin, pg/mL | -0.359 | -0.001 | (-0.003 to 0.001) | 0.391 | 0.428 | 0.001 | (-0.001 to 0.002) | 0.303 |
| BDNF, pg/mL | -0.271 | -0.001 | (-0.003 to 0.002) | 0.563 | 0.322 | 0.000 | (-0.001 to 0.002) | 0.487 |
| Myostatin, pg/mL | 0.059 | 0.000 | (-0.003 to 0.004) | 0.867 | -0.363 | -0.001 | (-0.004 to 0.001) | 0.304 |
| IGF-1, ng/mL | -0.374 | -0.072 | (-0.253 to 0.108) | 0.393 | -0.038 | -0.005 | (-0.127 to 0.117) | 0.929 |
| IGFBP-3 mg/L | 0.229 | 3.203 | (-8.773 to 15.179) | 0.564 | 0.026 | 0.249 | (-7.854 to 8.352) | 0.947 |

Values in bold mean statistical difference.

**Supplementary Table S3. Pearson correlation coefficient and multiple linear regression analyses examining functional performance, cytokines, adipokines, myokines, and health-related markers in participants with obesity plus LALM.**

|  | **5x-Chair stand, sec** | | | | **Handgrip strength, kg** | | | |
| --- | --- | --- | --- | --- | --- | --- | --- | --- |
| *Pearson correlation* |  | ***r*** | **P** |  |  | ***r*** | **P** |  |
| GDF-15, pg/mL |  | 0.048 | 0.425 |  |  | -0.006 | 0.490 |  |
| IL-6, pg/mL |  | 0.096 | 0.352 |  |  | 0.104 | 0.336 |  |
| Adiponectin, pg/mL |  | -0.382 | 0.059 |  |  | 0.195 | 0.212 |  |
| TNF-alfa, pg/mL |  | 0.324 | 0.095 |  |  | 0.157 | 0.260 |  |
| Resistin, pg/mL |  | -0.354 | 0.075 |  |  | 0.594 | **0.004** |  |
| BDNF, pg/mL |  | 0.172 | 0.248 |  |  | 0.258 | 0.143 |  |
| Myostatin, pg/mL |  | 0.204 | 0.209 |  |  | -0.181 | 0.229 |  |
| IGF-1, ng/mL |  | -0.053 | 0.418 |  |  | 0.195 | 0.212 |  |
| IGFBP-3 mg/L |  | 0.112 | 0.334 |  |  | 0.189 | 0.226 |  |
| *Multiple linear regression* | **ß** | **B** | **(95% CI)** | **P** | **ß** | **B** | **(95% CI)** | **P** |
| GDF-15, pg/mL | 0.177 | 0.001 | (-0.004 to 0.007) | 0.552 | 0.258 | 0.001 | (-0.003 to 0.005) | 0.479 |
| IL-6, pg/mL | -1.029 | -0.159 | (-0.311 to -0.008) | **0.042** | 0.128 | 0.012 | (-0.101 to 0.125) | 0.807 |
| Adiponectin, pg/mL | -0.240 | -2.677^e-5^ | (0.000 to 0.000) | 0.302 | 0.323 | 2.243^e-5^ | (0.000 to 0.000) | 0.256 |
| TNF-alfa, pg/mL | 1.441 | 3.066 | (0.819 to 5.312) | **0.015** | 0.136 | 0.180 | (-1.494 to 1.854) | 0.810 |
| Resistin, pg/mL | -0.669 | -0.001 | (-0.002 to 0.000) | **0.021** | 0.599 | 0.001 | (0.000 to 0.001) | 0.063 |
| BDNF, pg/mL | 0.137 | 0.000 | (-0.001 to 0.002) | 0.588 | 0.221 | 0.000 | (-0.001 to 0.001) | 0.476 |
| Myostatin, pg/mL | -0.363 | -0.002 | (-0.008 to 0.003) | 0.399 | -0.244 | -0.001 | (-0.005 to 0.003) | 0.635 |
| IGF-1, ng/mL | -0.892 | -0.195 | (-0.419 to 0.028) | 0.077 | 0.249 | 0.034 | (-0.132 to 0.200) | 0.650 |
| IGFBP-3 mg/L | 0.524 | 5.844 | (-3.152 to 14.839) | 0.168 | -0.076 | -0.530 | (-7.232 to 6.173) | 0.860 |

Values in bold mean statistical difference.

**Supplementary Table S4. Multiple linear regression analyses examining cytokines, adipokines, myokines, health-related markers and body composition parameters in the total sample.**

|  | **Lean Mass, Kg** | | | | **Fat, Kg** | | | |
| --- | --- | --- | --- | --- | --- | --- | --- | --- |
| *Multiple linear regression* | **ß** | **B** | **(95% CI)** | **P** | **ß** | **B** | **(95% CI)** | **P** |
| GDF-15, pg/mL | 0.343 | 0.002 | (0.001 to 0.003) | **0.004** | 0.309 | 0.003 | (0.001 to 0.005) | **0.009** |
| IL-6, pg/mL | -0.021 | -0.002 | (-0.029 to 0.025) | 0.885 | -0.030 | -0.005 | (-0.051 to 0.041) | 0.836 |
| Adiponectin, pg/mL | -0.089 | -5.714^e-6^ | (0.000 to 0.000) | 0.456 | -0.103 | -1.124^e-5^ | (0.000 to 0.000) | 0.386 |
| TNF-alfa, pg/mL | -0.017 | -0.014 | (-0.242 to 0.215) | 0.904 | -0.137 | -0.186 | (-0.572 to 0.200) | 0.340 |
| Resistin, pg/mL | 0.172 | 0.000 | (0.000 to 0.001) | 0.151 | 0.159 | 0.000 | (0.000 to 0.001) | 0.179 |
| BDNF, pg/mL | -0.049 | 0.000 | (-0.001 to 0.000) | 0.698 | 0.050 | 0.000 | (-0.001 to 0.001) | 0.689 |
| Myostatin, pg/mL | 0.084 | 0.000 | (-0.001 to 0.001) | 0.531 | 0.114 | 0.001 | (-0.001 to 0.003) | 0.390 |
| IGF-1, ng/mL | 0.030 | 0.005 | (-0.048 to 0.058) | 0.848 | -0.182 | -0.053 | (-0.142 to 0.037) | 0.243 |
| IGFBP-3 mg/L | 0.171 | 1.593 | (-1.134 to 4.320) | 0.248 | 0.124 | 1.965 | (-2.643 to 6.573) | 0.398 |
|  | **ALM, Kg** | | | | **Fat, %** | | | |
| *Multiple linear regression* | **ß** | **B** | **(95% CI)** | **P** | **ß** | **B** | **(95% CI)** | **P** |
| GDF-15, pg/mL | 0.339 | 0.001 | (0.0004 to 0.002) | **0.005** | 0.268 | 0.001 | (0.000 to 0.002) | **0.025** |
| IL-6, pg/mL | 0.005 | 0.000 | (-0.015 to -0.016) | 0.974 | -0.023 | -0.002 | (-0.026 to 0.023) | 0.877 |
| Adiponectin, pg/mL | -0.085 | -3.072^e-6^ | (0.000 to 0.000) | 0.483 | -0.033 | -1.910^e-6^ | (0.000 to 0.000) | 0.782 |
| TNF-alfa, pg/mL | -0.043 | -0.019 | (-0.150 to 0.111) | 0.769 | -0.122 | -0.086 | (-0.292 to 0.119) | 0.405 |
| Resistin, pg/mL | 0.137 | 0.000 | (0.000 to 0.000) | 0.258 | 0.061 | 7.716^e-5^ | (0.000 to 0.000) | 0.608 |
| BDNF, pg/mL | -0.004 | -5.397^e-6^ | (0.000 to 0.000) | 0.973 | 0.198 | 0.000 | (0.001 to 0.001) | 0.120 |
| Myostatin, pg/mL | 0.068 | 0.000 | (0.000 to 0.001) | 0.617 | -0.015 | -5.409^e-5^ | (-0.001 to 0.001) | 0.910 |
| IGF-1, ng/mL | -0.039 | -0.004 | (-0.034 to 0.027) | 0.804 | -0.230 | -0.035 | (-0.083 to 0.013) | 0.147 |
| IGFBP-3 mg/L | 0.170 | 0.888 | (-0.670 to 2.447) | 0.259 | -0.032 | -0.269 | (-2.723 to 2.186) | 0.828 |

Values in bold mean statistical difference.
